# Sensitization of glioblastoma tumor micro-environment to chemo- and immunotherapy by Galectin-1 intranasal knock-down strategy

**DOI:** 10.1038/s41598-017-01279-1

**Published:** 2017-04-27

**Authors:** Matthias Van Woensel, Thomas Mathivet, Nathalie Wauthoz, Rémi Rosière, Abhishek D. Garg, Patrizia Agostinis, Véronique Mathieu, Robert Kiss, Florence Lefranc, Louis Boon, Jochen Belmans, Stefaan W. Van Gool, Holger Gerhardt, Karim Amighi, Steven De Vleeschouwer

**Affiliations:** 1 Research Group Experimental Neurosurgery and Neuroanatomy, KU Leuven, Herestraat 49, Leuven, 3000 Belgium; 2 Laboratoire de Pharmacie Galénique et de Biopharmacie, Université libre de Bruxelles (ULB), Boulevard du triomphe CP207, Brussels, 1050 Belgium; 3 Vascular Patterning Laboratory, VIB Center for Cancer Biology, VIB, Leuven, Belgium; 4 Vascular Patterning Laboratory Department of Oncology, KU Leuven, Herestraat 49 box 912, Leuven, 3000 Belgium; 5 Cell Death Research & Therapy (CDRT) Lab, Department of Cellular and Molecular Medicine, KU Leuven, Herestraat 49, Box 802, Leuven, 3000 Belgium; 6 Laboratoire de Cancérologie et de Toxicologie Expérimentale, Université libre de Bruxelles (ULB), Boulevard du triomphe CP205/01, Brussels, 1050 Belgium; 7 Department of Neurosurgery, Erasmus University Hospitals, Route de Lennik 808-1070, Brussels, 1050 Belgium; 8 EPIRUS Biopharmaceuticals, BV, Yalelaan 46, Utrecht, 3584 The Netherlands; 9 Laboratory of Pediatric Immunology, KU Leuven, Herestraat 49 box 811, Leuven, 3000 Belgium; 10 Medizinische Leitung der Translationalen Onkologie, Immunologisches Onkologisches Zentrum, Köln, Germany; 11 Department of Neurosurgery, University Hospitals Leuven, Herestraat 49 box 7003, Leuven, 3000 Belgium

## Abstract

In this study, we evaluated the consequences of reducing Galectin-1 (Gal-1) in the tumor micro-environment (TME) of glioblastoma multiforme (GBM), via nose-to-brain transport. Gal-1 is overexpressed in GBM and drives chemo- and immunotherapy resistance. To promote nose-to-brain transport, we designed siRNA targeting Gal-1 (siGal-1) loaded chitosan nanoparticles that silence Gal-1 in the TME. Intranasal siGal-1 delivery induces a remarkable switch in the TME composition, with reduced myeloid suppressor cells and regulatory T cells, and increased CD4+ and CD8+ T cells. Gal-1 knock-down reduces macrophages’ polarization switch from M1 (pro-inflammatory) to M2 (anti-inflammatory) during GBM progression. These changes are accompanied by normalization of the tumor vasculature and increased survival for tumor bearing mice. The combination of siGal-1 treatment with temozolomide or immunotherapy (dendritic cell vaccination and PD-1 blocking) displays synergistic effects, increasing the survival of tumor bearing mice. Moreover, we could confirm the role of Gal-1 on lymphocytes in GBM patients by matching the Gal-1 expression and their T cell signatures. These findings indicate that intranasal siGal-1 nanoparticle delivery could be a valuable adjuvant treatment to increase the efficiency of immune-checkpoint blockade and chemotherapy.

## Introduction

Central nervous system neoplasms are subtyped in either primary or secondary brain tumors. Among primary brain tumors, glioblastoma multiforme (GBM) is the most prevalent high grade glioma^1, 2^. GBM displays highly necrotic, hypoxic and mitotic areas, hallmarks of high grade neoplasms^3^. Current therapy consists of debulking, followed by chemoradiotherapy, resulting in a median survival of 14.6 months^4^. Novel clinical treatment regimens have so far had little impact on GBM patient survival^5^. The field of immunotherapy offers promising new avenues^6^ with dendritic cell (DC) vaccinations^7–10^ or recent clinical trials targeting Programmed Cell Death protein-1 (PD-1) in order to regulate the immune checkpoint in GBM (ClinicalTrials.gov identifier: NCT02335918, NCT02529072). Targeting PD-1 aims to release the break on the adaptive immune response against tumour cells, leading to increased recruitement and activation of cytotoxic T cells (CTLs).

Despite numerous clinical trials, and novel biological compounds being tested, the poor prognosis for GBM patients remains largely unchanged^11, 12^. The uniquely immune-priviledged microenvironment of the central nervous system proves to be particularly challenging for the efficacy of immunotherapy in GBM. Therefore, the development of new therapies with improved activity at the tumor site will require a deeper understanding of the dynamic tumor micro environment (TME) in GBM. Recent findings identified a pivotal tumour promoting role for Galectin-1 (Gal-1), a 14 kDa lactose binding lectin, in the TME of GBM^13, 14^. Gal-1 increases the migration of GBM cells and their resistance against chemotherapy (i.e. temozolomide, TMZ)^15–17^. Gal-1 has further been correlated with the proliferative gain-of-function properties of tumor cells involving anchoring the RAS proto-oncogene at the innerplasmatic membrane^13, 18^. In addition, immunosuppressive and angiogenic properties of Gal-1 have been suggested in the context of GBM^17, 19^. Rubinstein *et al*. noticed that blockade of Gal-1 synthesis in tumor cells allows the generation of a tumor-specific Th1-type immune response *in vivo* ^20^. Interestingly, Gal-1 can be upregulated in tumors during radiotherapy, thereby sustaining immune suppression^21^. These features pinpoint Gal-1 as a crucial molecule in shaping the TME in GBM. Indeed, intraventricular delivery of siGal-1 can sensitize GBM cells to TMZ while reducing the abnormal tumour vasculature^17^. However, the invasive procedure of intraventricular administration is associated with inherent adverse events such as inflammation.

As a non-invasive alternative route to deliver therapeutic agents into the central nervous system, and more specifically into the TME of GBM, we proposed the nose-to-brain transport via intranasal administration^22^. In recent years, the intranasal transport towards the central nervous system has gained a lot of momentum, and even reached the clinical stage because of patient comfort and reduced adverse secondary effects^23–25^. Previously we described that chitosan nanoparticles are able to transport siGal-1 to the TME in GBM^26^. This leads to a specific degradation of Gal-1, while other galectines (such as Gal-3) are not affected. After administriation in the nasal cavity, siGal-1 is rapidly transported to the olfactory bulbus and spread within the inoculated glioma TME. Functionally, we observed a strong reduction of Gal-1 after siGal-1 treatment and subsequent cleaved Gal-1 mRNA fragments in GL261 glioma tumors. As such, we were able to provide strong evidence for *in vivo* RNAi mediated targeted reduction of Gal-1 via the intranasal pathway.

Here, we investigated the consequences of reducing Gal-1 in the TME during the GBM progression on both myeloid and lymphoid compartments of the immune system. Moreover, we evaluated the effect of Gal-1 siRNA therapy alone or in combination with chemotherapy or immunotherapy using DC vaccination and PD-1 blockade on survival of glioma bearing mice. The relevance of our findings were confirmed by the comparison of the Gal-1 knock-down signatures in our GBM model with GBM patients data from The Cancer Genome Atlas (TCGA) database. Our findings identify Gal-1 as a key regulator of the TME that drives resistance towards conventional chemotherapy and explorative immunotherapy. Nose-to-brain delivery to modulate the TME is an underexplored treatment modality and beholds great promise for GBM patients, especially when combined with chemo- and immunotherapy.

## Results

### siGal-1 treatment improves survival in GL261 tumor bearing mice by altering the tumor micro-environment

**Figure 1:**
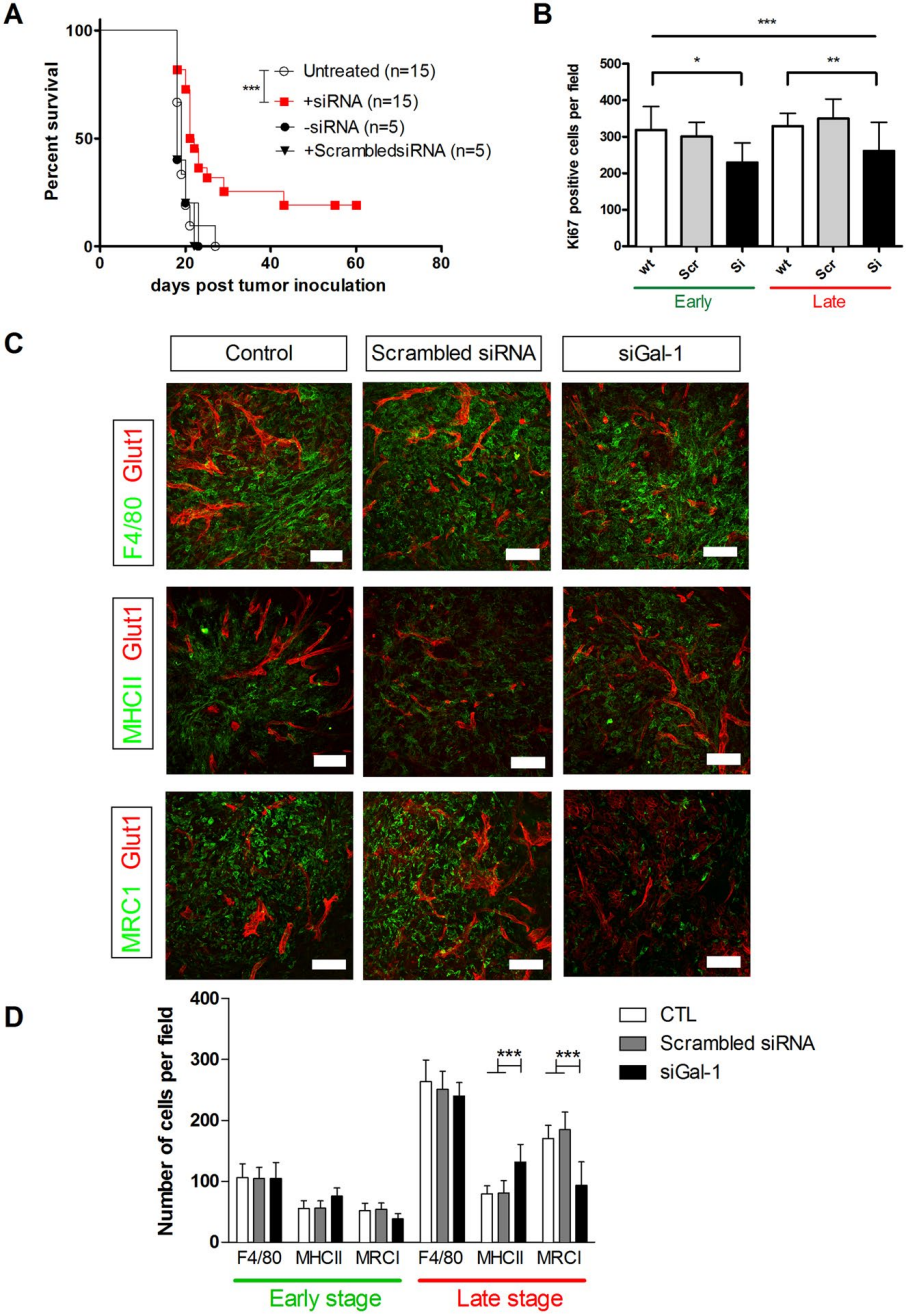
siGal-1 monotherapy in GL261 tumor bearing mice elicits a survival benefit, and a re-arrangement of tumor micro-environment. Mice were inoculated with 0.5 × 10^6^ GL261 cells which induce a lethal GBM after 15–20 days. (**A**) Survvial analysis of mice which were left untreated (clear dots), treated with siGal-1 on day 4, 8, 12 and 15 after tumor inoculation (red squares), or treated with empty nanoparticles (without siRNA, black dots) or treated with scrambled siRNA loaded chitosan nanoparticles (scrambled siRNA, black triangles). (**B**) Quantification of proliferation assay measured by Ki67+ staining. Monotherapy siGal-1 revealed a significant Ki67+ proliferation decrease in GL261 gliomas, in early and late stage (early stage = day 12 after tumor inoculation; 2 administrations, at day 4 and 8/at late stage GBM = day 20 after tumor inoculation, with 4 administrations at day 4, 8, 12 and 15), as compared to controls, untreated or scrambled siRNA loaded chitosan nanoparticles (n = 4/group, *p < 0.05 and ***p < 0.001). (**C**) Immunofluorescence pictures of TME, stained for total macrophages (F4/80+), M1 macrophages (MHCII+) and M2 macrophages (MRC1+), obtained from untreated control mice, scrambled siRNA loaded chitosan nanoparticles treated mice, or siGal1 treated mice. These pictures indicate that the total amount of macrophages in the TME is not affected, but merely the polarization of M1 to M2 polarization. MRC1+M2 macrophages are reduced with siGal-1 therapy, as compared to untreated or scrambled siRNA treated mice. (**D**) Quantification of multiple sections of several mice (n = 4/group), indicates the effect is most pronounced at late stage GBM (day 20 after tumor inoculation, with 4 administrations at day 4, 8, 12 and 15), whereas in early stage (day 12 after tumor inoculation; 2 administrations, at day 4 and 8) these differences were less pronounced. Black bars represent treated mice, white bars are untreated controls, and gray bars are treated with scrambled siRNA loaded chitosan nanoparticles; groups were compared via two way ANOVA, with Bonferroni post test as compared to control and scrambled siRNA (n = 4/group, ***p < 0.001). Scale bar: 75 µm.

To confirm our previous findings, we first verified the reduction of Gal-1 after siGal-1 administration (Supp. Fig. 1). Gal-1 is reduced whithin the tumor micro-environment by about 40%. In previously published results we observed that reducing *gal-1* expression can inhibit the migration and/or proliferation pattern of GL261 murine GBM cells *in vitro* ^26^. Stimulated by the existing literature on siGal-1 in GBM, we investigated possible survival benefits in GL261 tumor bearing mice. First, we asked whether intranasal siGal-1 as monotherapy could improve survival (+siRNA, Fig. 1A). We observed a significant shift in survival when siGal-1 loaded chitosan nanoparticles were administered during GBM progression. A small shift of 2.5 days in median survival, and more importantly, about 20% long-term survival was induced. Scrambled siRNA incorporated in chitosan nanoparticles or empty chitosan nanoparticles (+scrambled siRNA and -siRNA, Fig. 1A) had no effect on survival. To further delineate the consequences of siGal-1 treatment, we hypothesized that the prolonged survival could be explained either by a direct decreased proliferation of GL261 tumor cells, or by complementary TME re-arrangement. We first quantified Ki67+ staining to measure tumor proliferation. Proliferation was significantly decreased during siGal-1 treatment, while scrambled siRNA loaded chitosan nanoparticles had no effect on GL261 (Fig. 1B, representative pictures Supp Fig. 2). Of note, the inhibitory effect was already pronounced at day 12 post tumor inoculation, indicating a rapid delivery and onset of siGal-1. Caspase3 staining revealed no changes in apoptosis (Supp Fig. 3). To analyse the cellular composition of GL261 TME, a thorough examination of the macrophage populations was performed (Fig. 1C). Macrophages are one of the most abundant cell types present in the TME of GBM. They can be polarized as either M1 (MHCII^high^) or M2 (MRC^high^) corresponding to pro-inflammatory and anti-inflammatory phenotypes, respectively^27^. Gal-1 siRNA treatment induced a shift in these macrophage phenotypes: reduction of Gal-1 did not affect the total pool of macrophages, but effectively reduced the transition from M1 to M2 polarization (Fig. 1D). Scrambled siRNA did not affect macrophage polarization.

### siGal-1 treatment modulates myeloid and lymphoid cells during GBM progression

**Figure 2:**
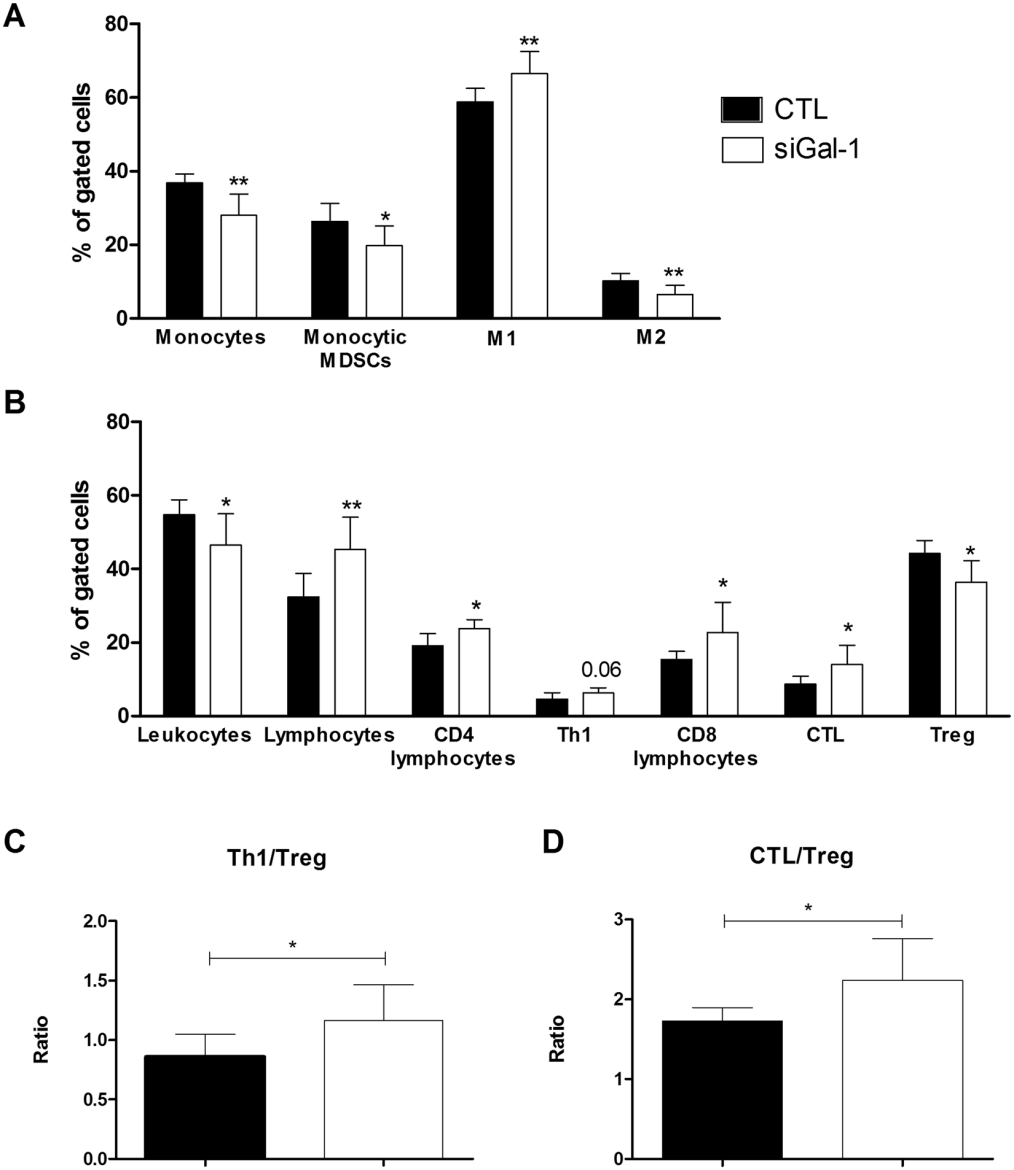
siGal-1 alleviates immune suppression and induces immune activation during GBM progression. Flow cytometry was performed on isolated mononuclear brain infiltrating cells of mice that were left untreated, or treated with siGal-1 on day 4, 8, 12 and 15 after tumor inoculation, and brains were isolated at day 20. Different stainings assess several cell populations with (**A**) the myeloid cells, with monocytes as gated by ZY−, CD45+, CD11b+; monocytic MDSCs as ZY−, CD45+, CD11b+, Ly6C+; M1 macrophage phenotype as CD45+, CD11b+, ZY−, MRC−, MHCII^high^; M2 macrophage phenotype as CD45+, CD11b+, ZY−, MRC+, MHCII^low^. (**B**) The lymphoid cells with leukocytes, as single cells, ZY−, CD45+; lymphocytes as single cells, ZY−, CD45+, CD3+, CD4 lymphoctyes as single cells, ZY−, CD45+, CD3+, CD4+; Th1 as single cells, ZY−, CD45+, CD3+, CD4+, IFNγ+ gated to CD45+; CD8 lymphocytes as single cells, ZY−, CD45+, CD3+, CD8+; CTL as single cells, ZY−, CD45+, CD3+, CD8+, IFNγ+ gated to CD45; Tregs, as gated by single cells, ZY−, CD45+, CD3+, CD4+, FoxP3+; (**C**) the ratio immune activation to immune suppression was calculated for Th1 (IFNγ+CD4+CD3+CD45+ZY−) and (**D**) CTL (IFNγ+CD8+CD3+CD45+ZY−), as compared to Treg (FoxP3+CD4+CD3+CD45+). White bars represent the siGal-1 treated mice, black bars are control tumor bearing mice and groups were compared with unpaired t-test (n = 5/10 per group, *p < 0.05, **p < 0.01).

We next assessed whether Gal-1 expression is linked to changes in the myeloid compartment of TME infiltrating leukocytes. Using flow cytometry, we observed a reduction in CD11b+ leukocytes from 37 to 28% in response to siGal-1 intranasal administration (Fig. 2A; gating strategy Supp Fig. 4). Further subtyping revealed that this reduction was mainly caused by a reduction in immune suppressive monocytic myeloid derived suppressor cells (MDSCs) defined as Ly6C+CD11b+ leukocytes from 26 to 20%. The percentage of granulocytic MDSCs (Ly6G+CD11b+) appeared unaffected. Besides MDSCs in the TME, also macrophages can exert immune suppressive functions. As described above, macrophages can be subtyped in M1 (MHCII^high^, MRC−) or M2 (MHCII^low^,MRC+) of which the latter are known to reduce inflammation and negatively affect the prognosis in GBM. Flow cytometry analysis confirmed that the reduction of Gal-1 promotes the M1 phenotype, and prevents the M2 phenotype (Fig. 2A).

As the immune suppressive myeloid cells compartment was altered by Gal-1 siRNA treatment, we analysed the lymphoid counterpart and monitored the regulatory T cell population (Treg). Both at the mRNA and protein level, we detected a decrease of FoxP3+ cells, a Treg specific transcription factor (Fig. 2B + Supp Fig. 5; gating strategy Supp Fig. 6). Together these data indicate a general reduction of immunosuppression in GBM, in both the innate and adaptive arm of immunity, when *gal-1* expression is diminished.

On the other side of the spectrum, we have investigated the immune stimulatory mediators in GBM. Overall, we found a decrease in leukocytes (CD45 + viable cells; Fig. 2B; gating strategy Supp Fig. 7), which we attribute to the decrease in overall myeloid MDSCs. Further co-staining revealed a significant increase in CD3+ lymphocytes (Fig. 2B). Specifically, we found a general increase in T helper lymphocytes (Th, CD4+CD3+lymphocytes), and cytotoxic T cells (CTL, CD8+CD3+lymphocytes). These cells appeared not only increased in number, but also in function, as measured by their interferon–gamma (IFNγ) production, indicated as Th1 (CD4+) and CTL (CD8+) lymphocytes relative to the total amount of leukocytes (Fig. 2B). As we observed an increase in Th1 and CTL response, and a decrease in Treg population, the ratio of immune activation to immune suppression was clearly shifted in favor of activation (Fig. 2C+D). Taken together, these data indicate that siGal-1 treatment can induce an alleviation from the immune suppression, while increasing the immune activation.

### siGal-1 therapy synergizes with TMZ and PD-1 blocking

**Figure 3:**
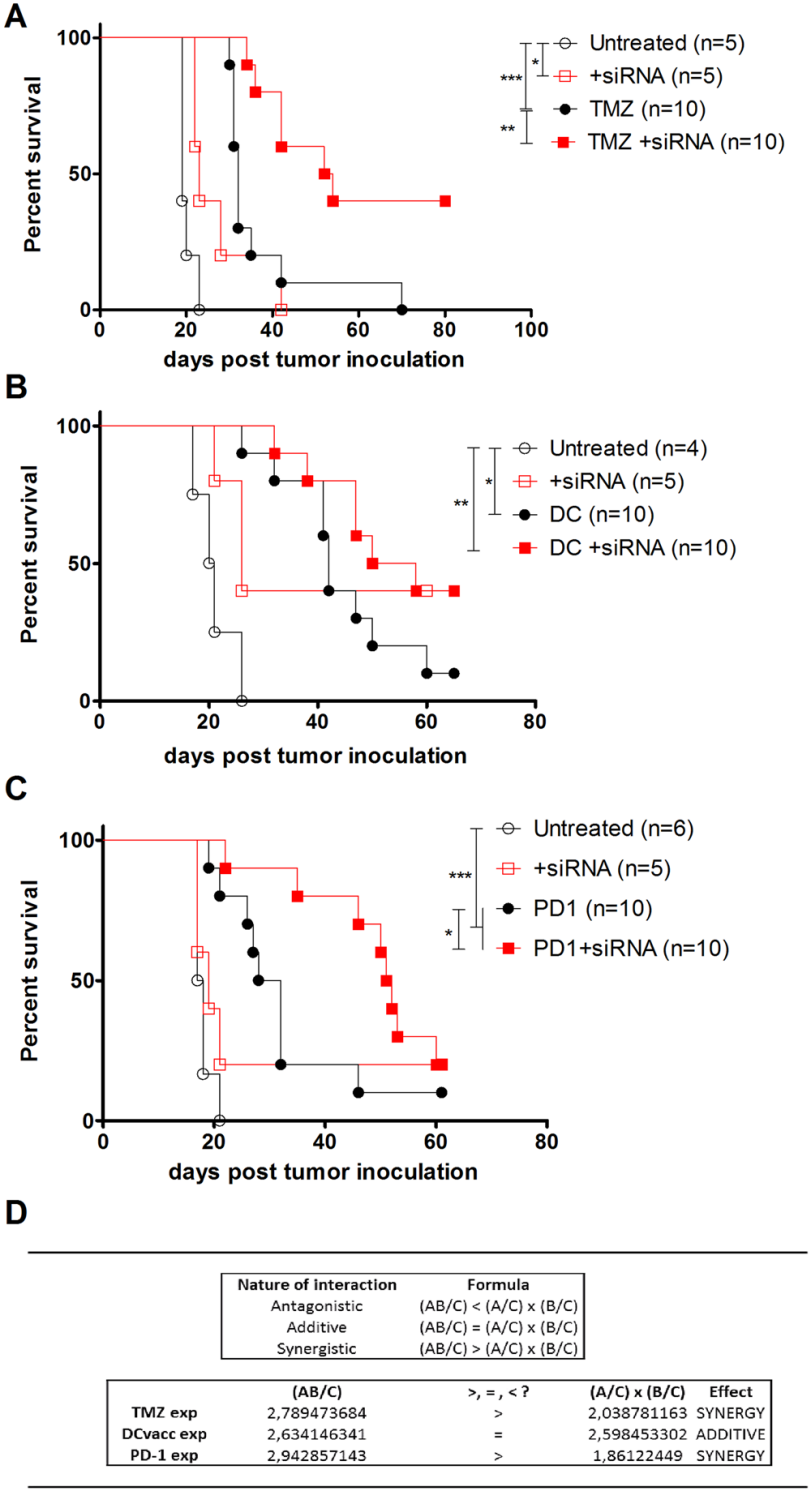
Survival benefit of siGal-1 in combination with chemo- and immunotherapy. Mice were inoculated with 0.5 × 10^6^ GL261 cells which induce a lethal GBM after 15–20 days. Combination strategy with (**A**) TMZ was organized by siGal-1 administration at day 2, 4, 7 and 11 after tumor inoculation prior to TMZ administration at day 8, 11 and 15, at a dose of 40 mg/kg. For combination with immunotherapy, DC vaccination (**B**) and PD-1 blocking (**C**) were included. In (**B**), mice received monotherapy siGal-1 as described in Fig. 1A (open red squares), prophylactic DC vaccination at day −14 and −7 before tumor inoculation (200 µg lysate/million DCs, IP, black dots), or the combination of DC vaccination and siGal-1 (closed red squares). In (**C**), mice received anti-PD-1 mAb at day 7 and 12 after tumor inoculation (100 µg/day, IP, black dots) or the combination of PD-1 blocking and siGal-1 (closed red squares). Curves were compared by Log-Rank survival analysis. (n = as indicated, *p < 0.05, **p < 0.01 and ***p < 0.001). (**D**) Based on median survival data (from Fig. 3A,B and C), the calculations for antagonistic effect, additive effect and synergistic effect were carried out based on the Robert Clark Equations^29^; where A = response to treatment 1 (=siGal-1), B = response to treatment 2 (=TMZ, DCvacc or anti-PD-1), AB = combination groups and C = response to no treatment (=control).

To our understanding, siGal-1 treatment should be applied as an adjuvant therapy that synergizes with existing treatment schedules. As mentioned before, reducing *gal-1* expression has been shown to improve the efficacy of chemotherapeutic drugs such as TMZ in GBM bearing mice^17^. Also in our GL261 model, after three administrations of 40 mg/kg, we observed a significant median survival shift for glioma bearing mice, from 19 days in untreated mice, to 32 days in TMZ treated mice (Fig. 3A). Combining the intranasal siGal-1 administration and per os TMZ administration resulted in a synergistic survival benefit. In the combination therapy, the median survival increased from 32 days in TMZ treated mice, to 53 days in siGal-1 and TMZ treated mice. We also observed a long-term survival of 40% after combination therapy, compared to about 20% under monotherapy. These results indicate that reducing *gal-1* expression could be applied as an adjuvant treatment modality to TMZ administration.

Given that reducing *gal-1* expression alone could shift the balance from immune suppression to activation, resulting in a modest survival benefit (Fig. 1A), combining siGal-1 with other immunotherapies seemed appealing. Therefore we started with a prophylactic vaccination model established in our facilities^28^. Mice were vaccinated two weeks before tumor inoculation with lysate pulsed DC vaccines which results in strong anti-tumoral immunity as indicated by the survival benefit of 20.5 days in untreated mice, to 42 days in DC vaccine treated mice (Fig. 3B). We observed that combining DC vaccine with siGal-1 further pushed the significance level, and increased the median survival to 53 days with about 30% long-term survival. No statistical difference was observed between DC vaccine and the combination therapy, largely attributed to the heterogeneous effect of DC vaccine. As a second immunotherapy approach, we tested immune checkpoint blockade by means of PD-1 blocking in established brain tumors. Intraperitoneal injection of anti-PD-1 antibody increased the survival from 17.5 days in untreated mice, to 30 days in anti-PD-1 treated mice (Fig. 3C). Concomitant intranasal administration of siGal-1 profoundly improved the therapeutic effect of anti-PD-1 administration, resulting in a median survival of 51.5 days and about 20% long term survival.

To quantify the effect of the combination regimens with siGal-1, TMZ, DC vaccination and PD-1 blocking, we used the Robert Clarke Equations, that can discriminate between additive or synergistic effects (Fig. 3D)^29^. Calculation revealed an additive effect of DC vaccination and siGal-1 combination, and a synergistic effect of siGal-1 with either TMZ treatment or with PD-1 blocking antibody injection.

We conclude from these data that siGal-1 therapy is effective in a rational combination with chemo- and immunotherapy.

### Mechanistic insight into synergistic effects of siGal-1

**Figure 4:**
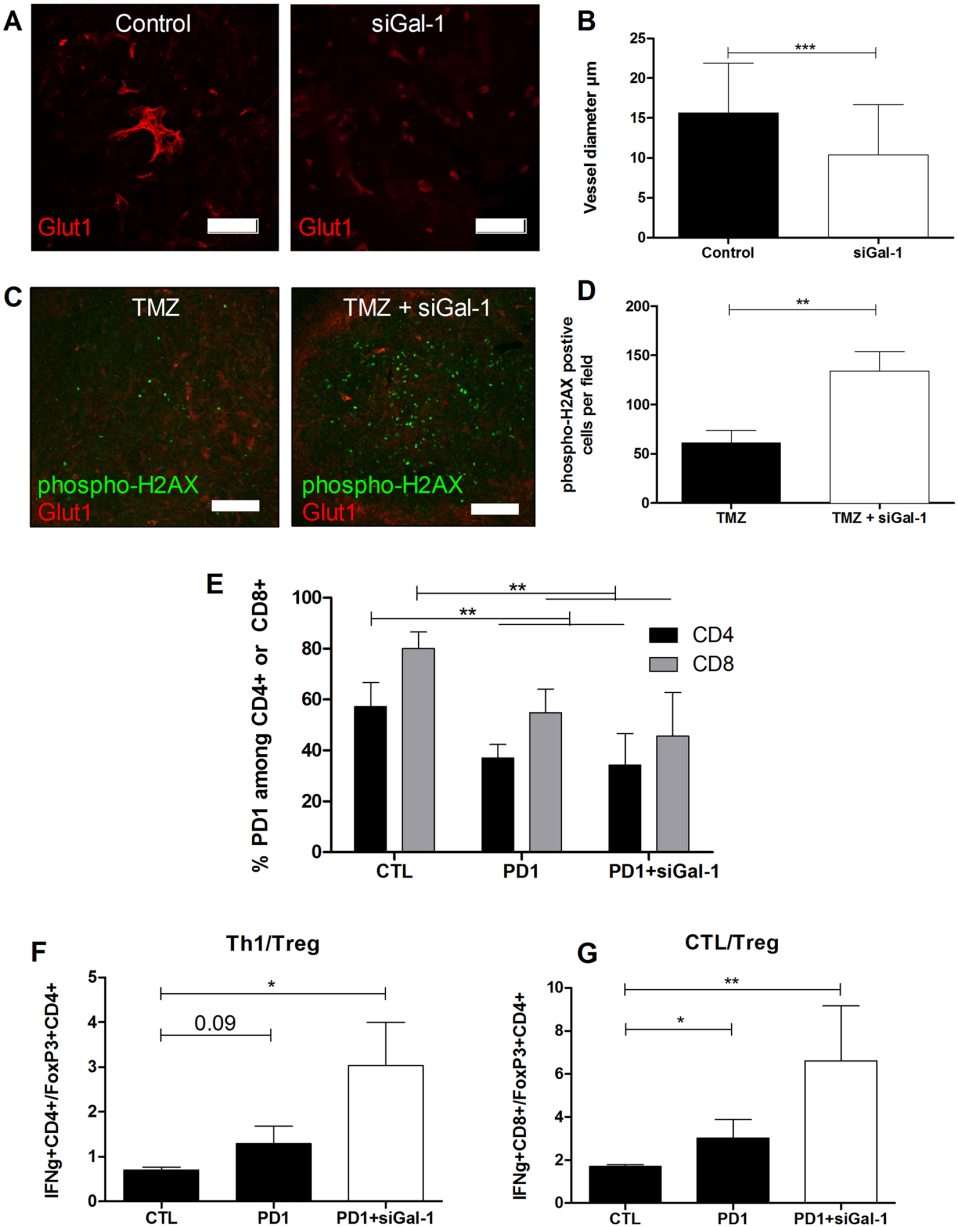
Mechanistic insights explaining synergistic effects of siGal-1 with chemo and immunotherapy. Mice were inoculated with 0.5 × 10^6^ GL261 cells which induce a lethal GBM after 15–20 days. (**A**) Mice were left untreated (left panel), treated with siGal-1 on day 4, 8, 12 and 15 after tumor inoculation (right panel), and stained for GLUT-1 (red), which stains vasculature. (**B**) Quantification revealed signficantly enlarged vasculature in untreated mice. (n = 5/group, **p < 0.01). (**C**) TMZ induced DNA damage was measured by phospho-H2AX staining (green), which (D) indicated a significant higher DNA damage pattern if mice were pre-treated with siGal-1. (n = 3/4/group, **p < 0.01). (**E**) Analysis of brain infiltrating lymphocytes during anti-PD-1 therapy (at day 7 and 12 after tumor inoculation, 100 µg/day), at day 18 post tumor inoculation, revealed a significant decrease of PD-1 expression on CD4+ (black bars) and CD8+ (gray bars) lymphocytes. In (**F**) the ratio immune activation to immune suppression was calculated for Th1 (IFNγ+CD4+CD3+CD45+ZY−) and CTL (**G**) (IFNγ+CD8+CD3+CD45+ZY−), as compared to Treg (FoxP3+CD4+CD3+CD45+). (n = 5/group, *p < 0.05 and **p < 0.01). Scale bar: A. 100 µm; C. 250 µm.

We observed a small survival benefit for mice that received monotherapy siGal-1 (Fig. 1A) and a strong synergistic effect of reducing *gal-1* expression in combination with chemo and immunotherapy. Therefore, we tried to elucidate the underlying mechanisms driving the synergy. We noticed that blood vessels in the tumor bearing mice that were treated with siGal-1 appeared less dilated and tortuous than in untreated mice (Fig. 4A). In untreated control mice, the tumor vasculature typically features dilated and non functional vessels whereas *gal-1* targeting therapy significantly normalizes vessel caliber (Fig. 4B). Scrambled siRNA however does not normalize the vasculature in GBM (compare vessels in Fig. 1C). Chaotic TME blood vessels typically result in a poor tissue perfusion. We therefore hypothesized that TMZ could reach a larger tumor area if vessels were normalized to some extent^30^. To observe the distribution pattern of TMZ, we performed immunohistochemistry labelling phospho-H2aX to highlight double strand break DNA damage (Fig. 4C and D). Equal dose of TMZ induced more widespread DNA damage after pre-treatment with siGal-1, consistent with the observed normalization of the tumour vasculature.

To elucidate the synergy between siGal-1 and anti-PD1 therapy, we performed flow cytometry analysis of tumor infiltrating lymphocytes. We observed that anti-PD1 therapy effectively reduced the expression of PD-1 on CD4+ and CD8+ lymphocytes in the glioma TME (Fig. 4E), but no additional effects of combined siGal-1 treatment. Next, we observed that the ratio between Th1 response and Treg, or CTL and Treg, was increased upon anti-PD-1 therapy, and even more pronounced in combination therapy (Fig. 4F and G). These data, together with the survival benefits (Fig. 3C), indicate that anti-PD1 therapy by itself can stimulate the immune activation, and also that siGal-1 therapy can further increase the immune stimulation.

### *LGALS1* facilitates immunosuppressive T cell milieu in glioblastoma tumor tissue

Our preclinical results indicated that Gal-1 promotes immunosuppression within the GBM TME and as such, targeting it helps shift the TME towards an anti-tumorigenic Th1/CTL-based immune contexture. Therefore, we wanted to validate whether Gal-1 (coded by the gene *LGALS1*) also facilitates immunosuppression of T cells within the GBM TME of human patients. We deemed such analysis crucial for translational positoning and prioritization of this siGal-1 therapy for GBM patients in the near future.

High-powered genetic analysis of human tumor-associated T lymphocytes (TILs) in publicly available big-datasets like The Cancer Genome Atlas (TCGA) can be achieved through the emerging technique of using (pre-established) T cell-specific gene signatures or metagenes^31–33^. To this end, we utilized recently established T cell-specific metagenes tailored for human GBM^12^ and first estimated GBM patient survival based on a ratio of Th1-to-Treg and CTL-to-Treg to estimate the prognostic impact of a Th1/CTL vs. Treg based immune contexture.

**Figure 5:**
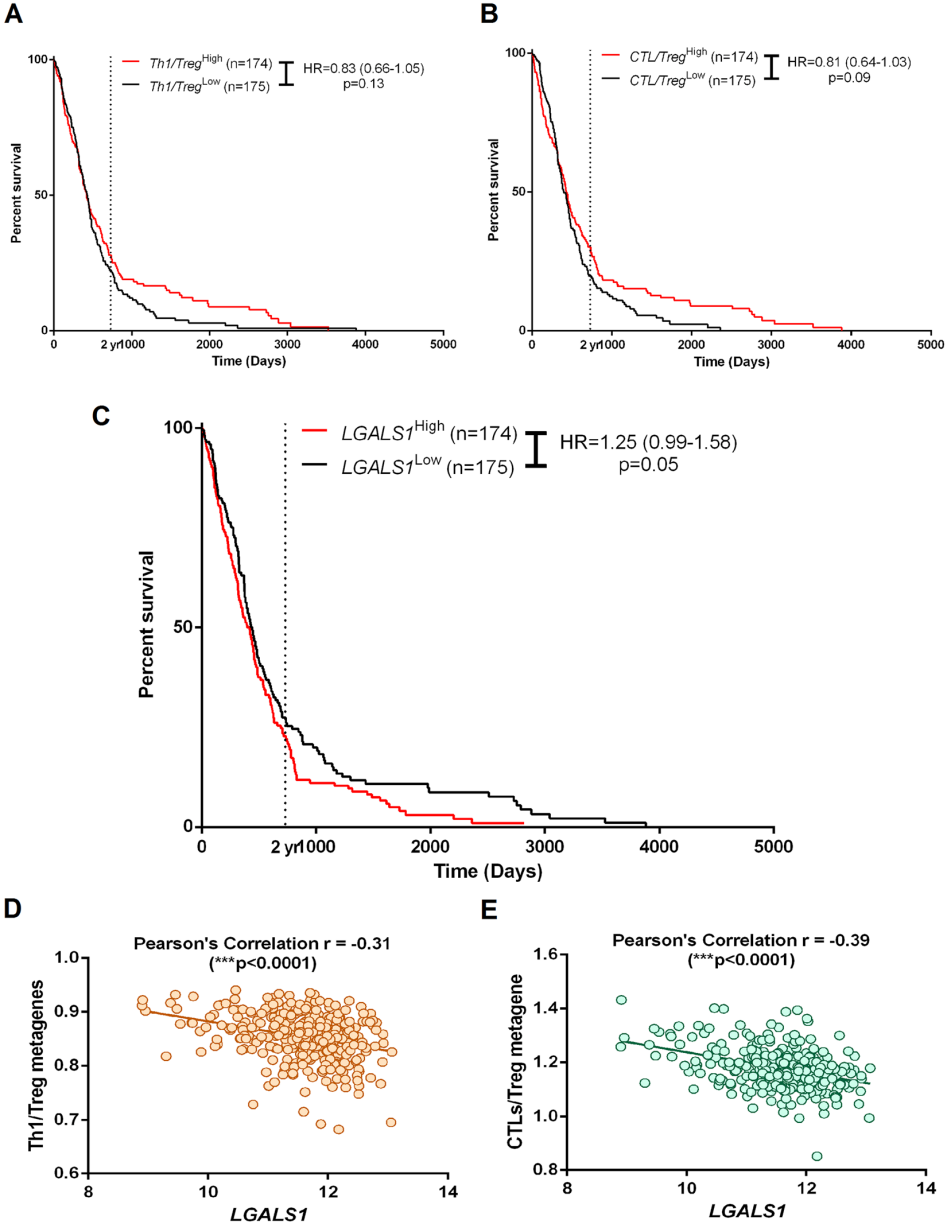
Analysis of association between T cell-associated genetic signatures and *LGALS1* in human glioblastoma (GBM) patients. (**A**) The ratio of genetic signatures or metagenes specific for Th1 and Treg was used to stratify the The Cancer Genome Atlas (TCGA) GBM patients into “high Th1/Treg ratio” (red) or “low Th1/Treg ratio” groups; followed by Kaplan-Meier plotting of patient’s overall survival versus follow-up duration in days. (**B**) The ratio of genetic signatures or metagenes specific for CTLs and Treg was used to stratify the TCGA GBM patients into “high CTL/Treg ratio” (red) or “low CTL/Treg ratio” groups; followed by Kaplan-Meier plotting of patient’s overall survival versus follow-up duration in days. (**C**) The differential expression of *LGALS1* was used to stratify the TCGA GBM patients into “high expression” (red) or “low expression” groups; followed by Kaplan-Meier plotting of patient’s overall survival versus follow-up duration in days. In **A–C** graphs, log-rank (Mantel-Cox) test p-values and hazard ratios (HR) with 95% confidence interval (in paranthesis) are displayed. Alternatively, a Pearson’s correlation coefficient was calculated to assess the overall association between *LGALS1* expression and overall expression of either Th1/Treg ratio (**D**) or CTL/Treg ratio (**E**).

In line with previous observations^12^, TCGA GBM patients exhibiting higher expression of Th1/Treg metagene-ratio (i.e. more Th1 expression and less Treg expression) (Fig. 5A) and CTL/Treg metagene-ratio (Fig. 5B), had prolonged overall survival (OS) compared to corresponding low expression groups, as indicated by hazard ratios (HRs; 0.83 (0.66–1.05) for Th1/Treg metagene-ratio and 0.81 (0.64–1.03) for CTL/Treg metagene-ratio). This reiterates the importance of a Th1/CTL-based immune contexture over a Treg-based immune contexture for cancer patients^12, 34^.

Interestingly, GBM patients exhibiting high expression of GBM-associated *LGALS1* tended to show subtantially reduced OS compared to patients possessing *LGALS1* ^Low^ GBM tumors (Fig. 5C). In line with previous studies^13, 35, 36^, these findings confirm (at the TCGA-level) our observation of Gal-1 being a pro-tumorigenic factor. However, Gal-1 has several functions within the tumor, not only in glioma cells but also in stromal or immune cells. With respect to our preclinical data and the clinical analysis above, it was necessary to ascertain the exact association of *LGALS1* with Th1/Treg and CTL/Treg ratios. Interestingly, *LGALS1* gene expression showed a highly significant negative correlation with the Th1/Treg (Fig. 5D) and CTL/Treg (Fig. 5E) metagene-ratios within the TCGA GBM cohorts. These results would be in line with a role for higher *LGALS1* expression facilitating the accumulation of Treg over Th1/CTL in GBM.

In conclusion, our TCGA GBM clinical data analysis suggests that *LGALS1* facilitates the maintenance of a immunosuppressive Treg-based tumoral environment.

## Discussion

Understanding the cellular and molecular dynamics in the TME of malignancies such as GBM is a prerequisite for the development of effective non-invasive therapies. Our current work focused on Gal-1 as an important driver for the inherent resistance against chemo- and immunotherapy in GBM biology. Specifically, we demonstrate that, by using the nose-to-brain transport, we are able to suppress *gal-1* expression in the TME, which results in a shift from immune suppression to immune activation. Our intranasal approach to block *gal-1* expression in GBM confirms the previous benefitial findings that were however obtained by much more invasive approaches not translatable to the clinic. Moreover, we could demonstrate synergistic effects with novel immunotherapeutic agents as PD-1 blocking agents, which gives additional arguments why nose-to-brain transport to reduce *gal-1* expression could represent a valuable adjuvant therapy. In the present study, we explored the potential of chitosan nanoparticles to promote the nose-to-brain delivery. In part, these particles were selected due to the ease of manufacturing, the gentle production process and the proven transport into the central nervous system. In a recent literature report, chitosan coated lipid nanocapsules have been proven to effectively silence Gal-1 and EGFR in GBM, which also sensitizes GBM to TMZ treatment^37^. To this end, both lipid-based and polymeric based formulations can have beneficial effects, and are demonstrated to posses effective RNAi delivery efficiency.

In a syngenic, orthotopic murine model for GBM, we provide evidence that reducing *gal-1* expression induces a significant survival benefit. We observe a shift in median survival, and a long term survival induction of 20%. Especially the induction of long term survivors is intriguing, which coincided with remarkable changes in the microenvironment. Moreover, we demonstrate a decreased Ki67 proliferation in siGal-1 treated GL261 gliomas. In a previous report, we demonstrated that we are able to decrease *gal-1* expression in the TME by 50% as measured by western blot analysis^26^. Despite this robust decrease of Gal-1, the survival benefit in monotherapy is only modest, which already implies that siGal-1 should be considered as a sensitization tool to increase the efficiency of existing treatment modalities. To analyse the cell composition of the GBM TME, we dissected the macrophage population during GBM progression. We demonstrate the inhibition of polarization from M1 macrophages to M2 macrophages during GBM progression, when treated with siGal-1. Activation of the M2 phenotype has been shown to worsen the prognosis and aggravate disease through secretion of vascular promoting factors such as VEGF-A, and immune suppressive factors such as TGF-β and IL-10. This macrophage population relocates around TME associated vessels to form perivascular cuffs and further drives vascular abnormalities (Mathivet T. *et al*.; Dynamic stroma reorganization drives blood vessel dysmorphia during glioma progression; under revision). Therefore, a decrease in M2, but also in CD11b monocytes, and specifically the monocytic MDSCs, can already relieve a major fraction of the immune suppressive nature of the TME^38^. In recent years, it has been suggested that there is an intensive crosstalk between the immune suppressive actors of the myeloid side and of the lymphoid arm of the immune system. We also find a decrease in FoxP3+ Treg cells in the TME when Gal-1 is reduced. Whether this effect is mediated by a myeloid-lymphoid interaction or a direct effect of Gal-1 remains unclear^39^. The presence of Gal-1 and the recruitment of FoxP3+ Treg cells has already been established in previous reports^40, 41^. Not only do we find evidence for alleviation of immune suppression upon siGal-1 treatment, but also for promoting immune activation. CD3+ lymphocytes are increased within the TME upon Gal-1 reduction, and more specifically, both Th1 and CTL infiltrate the GBM. It has been demonstrated that GL261 cells upon IFN-γ stimulus, can up regulate MHCI molecules, and thereby are more susceptible to CD8+ mediated destruction^12^. These findings are in line with the proposed role of Gal-1 that drives apoptosis in activated T cells when reaching the TME^42^.

In further functional testing of our intranasal siGal-1 approach, we do not consider siGal-1 therapy as a monotherapy, but rather as an adjuvant therapy, which can be combined with chemo- and immunotherapy. Combination therapy of intranasal siGal-1 (at a dose of 1 unit at day 2, 4, 7 and 11) and per os temozolomide (at a dose of 40 mg/kg at day 8, 11 and 15) elicits a significant synergistic survival benefit. The shift in median survival was similar to that described previously, with intratumoral and intraventricular siGal-1 injections^17^, although in previous studies no long-term survivors were observed, most likely due to the use of nude-mice, and therefore lacking an immune component. This underlines the bio-equivalence of the non-invasive intranasal pathway, in comparison with more aggressive, invasive therapies. We observed that treatment with siGal-1 can reduce the vascular diameter in the TME from 15 µm to 10 µm. Previous reports describe how tumor derived Gal-1 can enhance endothelial function in migration and proliferation potential^43^, or even complement VEGF signaling^44, 45^. This reduction can be either attributed to the reduction in M2 (and lack of VEGF-A secreted in the perivascular spaces), or to direct anti-angiogenic effects of siGal-1. Therefore, the pre-treatment with siGal-1 could improve TMZ delivery through better tumor vessel perfusion in the entire TME, thus reaching the tumor mass *in toto*. We provide a functional experimental read-out in showing aggrevated and widespread DNA damage, which is not necessarily the result of enhanced perfusion, but supports the hypothesis. On the other hand, the reduction of Gal-1 obtained with siGal-1 pre-treatment alters the unfolded protein response to endoplasmatic reticulum stress, increasing thereby the inherent sensitivity of GBM cells to TMZ delivered^17^. Accordingly, in other, non-reported experiments, where siGal-1 therapy was administered concomitantly or later in the dosing schedule, we did not observe this synergy. This dose regimen optimization supports the hypothesis that siGal-1 should be delivered prior to TMZ administration. Assessment of the aggravated DNA damage induced by alkylating chemotherapy in the combination demonstrates that more tumor tissue is affected by administration of TMZ, as pointed out by the increase of phospho-H2aX histone complexes. This increase results in G2/M arrest^46^. Interestingly, TMZ is also reported to have anti-angiogenic properties, which might attribute to the observed synergistic effects between siGal-1 and TMZ^47^. In this study, TMZ was discovered to impair angiogenic processes, leading to strong survival benefits of human xenograft models when combined with bevacizumab. Interestingly, in a recent clinical trial report, TMZ showed unexpected immune-modulating activity^48^. Low-dose regimen TMZ could actively suppress regulatory T cells patients with advanced melanoma. We therefore speculate that siGal-1 and TMZ can work together to actively decrease the regulatory T cell population, and prevent further tumor progression. Alternative combination treatments can be found in the field of immunotherapy. In a first approach, we tested a DC vaccination strategy developed in our laboratory, as a potent prophylactic immunotherapy in the murine GL261 model. In the tested dosing combination, we could only observe non-significant additive survival benefit upon Gal-1 reduction. However, with immune checkpoint inhibition via PD-1 blockade, combination therapy leads to a synergistic survival benefits in mice with established brain tumors. We also find an increased immune stimulation of lymphocytes in the TME in the combination treatment, as compared to PD-1 blocking alone. Of note, aberrant vasculature in tumors is suggested as a substantial barrier for the extravasation of T cells^38^. As siGal-1 could efficiently reduce the vascular abnormalities, this could potentially explain the influx of Th1 and CTL. According to Robert Clark Equations, we observed a synergistic effect of combining anti-PD-1 therapy and TMZ therapy with siGal-1, and an additive effect of DC mediated immunotherapy in combination with siGal-1 (Fig. 3D). Adjuvant therapies such as siGal-1 therapy might therefore represent valuable approaches to further increase the efficiency of anti-PD-1 and TMZ therapy.

To validate our findings, we correlated the murine data with their human TCGA patient counterpart. Also in human samples, we found a clear correlation between lower Gal-1 and immune activation. Patients with lower Gal-1 had a more favorable Th1/Treg or CTL/Treg balance, which reflects in a better prognosis, as demonstrated by hazard ratios. Murine models have many advantages, but also inherent limitations. By demonstrating the translatability of Gal-1 biology on T cells from murine to human samples, we confirm the potency of Gal-1 as a driver of immune suppression during GBM progression.

In our research, we have elaborated on several key features of GBM tumor progression that are driven by Gal-1 biology. We have addressed proliferation, angiogenic, immunological, and TMZ-resistance properties of GBM after targeting *gal-1* expression via intranasal siRNA delivery. This research is a first step to pave the way towards a clinical implementation of the nose-to-brain transport of siGal-1 in the current treatment schedule of GBM patients. To our knowledge, this is the first report where the aforementioned pathway is validated from pharmaceutical development, to biological efficacy in a GBM tumor model. Moreover, as PD-1 blocking is currently being tested in clinical trials for GBM, we provide a method to augment its efficacy via a combination with this transnasal siGal-1.

## Materials and Methods

### Animals and treatments

International ethical guidelines were followed and approved by the bioethics committee at KU Leuven. Tumor inoculations were performed as described previously^26^. In brief, 0.5 × 10^6^ GL261 cells were stereotactically injected in the striatum of 8–10 weeks old C57Bl/6 mice (C57BL/6JOlaHsd, Harlan,The Netherlands). GL261 cells were received as a gift from Dr. Eyupoglu, University of Erlangen, Germany. Long term survival was defined as exceeding 3 x median survival of untreated control mice. Intranasal siGal-1 administrations were administered as 8 droplets of 3 µl with a non-adhesive micropipette tip (Eppendorf). Total amount of 1 administration was defined by 48 µg siRNA/dose and chitosan nanoparticles composition as described before^26^. In brief, chitosan nanoparticles were prepared by ionic gelation of tripolyphosphate and chitosan, while adding siRNA. Consequently, particles were collected by ultracentrifugation at 40000 × *g* during 3 cycles of 20 min, and freeze-dried with sucrose as lyoprotectant. At the indicated time points, chitosan nanoparticles were administered intranasally, under 3% isoflurane anesthesia. Mice that received intranasal PBS (control) or chitosan nanoparticles, experienced shivers and extensive grooming after anesthesia, followed by a small weight loss (1–2 g) the next day, due to the isoflurane administration of typically about 30 min/mouse. This weight loss quickly recovered, although in the 3th week after tumor inoculation, mice start to loose weight due to tumor burden. Sequences: siGal-1 5′ACCUGUGCCUACACUUCAAdTdT3′) and scrambled siRNA (5′GGAAAUCCCCCAACAGUGAdTdT3′) were purchased from GE Dharmacon.

TMZ administrations were performed as described previously^12^. In brief, Temodal capsules were opened and dissolved in a phosphate buffer, with an equal amount of L-Histidine, and administered in a total volume of 200 µl by gavage (Schering-Plough, Belgium). For survival experiments mice received 40 mg/kg TMZ at day 8, 11 and 15 post tumor inoculation. For phospho-H2AX assessments, mice received 80 mg/kg TMZ at day 13 and 14 post tumor inoculations, and were sacrificed for immunostainings 4 h after the last administration.

DC vaccinations were performed as described previously^28^. In brief, 200 µg irradiated lysate was loaded per 1 × 10^6^ immature DCs. Subsequently DCs were pulsed towards mature DCs with LPS, settled for 24 h, and intraperitoneal administered at day 14 and day 7 prior to tumor inoculation. Anti-PD-1 antibodies (RMP1-14) and isotype controls (Rat IgG) were dissolved in saline (Braun, The Netherlands) and intraperitoneal administered at day 7 and 12 post tumor inoculation (100 µg/administration, Epirus Biopharmaceuticals).

### Flow cytometry

Flow cytometric analysis was performed as described earlier^12^. In brief, animals were sacrificed by lethal injection of Nembutal at the indicated time points, and perfused with PBS (Lonza, Belgium). Single cell suspensions were obtained after mincing with scalpels and 30′ incubation with DNase (Invitrogen) and CollagenaseD (Roche). Mononuclear cells were separated from debris via Percoll gradient centrifugation (Sigma), and the intermittent layer was washed twice with PBS. Surface stainings were performed with antibodies as mentioned in Table 1.

**Table 1:** Flow cytometry antibodies.

Antigen	Fluorochrome	Origin
CD45	AF700	Ebioscience
CD11b	BV421	BD
Ly6C	AF647	Biorad
Ly6G	FITC	BD
MHCII	PerCP Cy5.5	Biolegend
Mannose Receptor	PE	Biolegend
Isotype Rat IgG2a,k	PE	Biolegend
Live/dead	Zombie Yellow	Biolegend
CD3	FITC/PE	Ebioscience
CD4	PerCP Cy5.5/APC-eF780	Ebioscience
CD8	BV421	BD
NKp46	APC	Biolegend
FoxP3	PE	Ebioscience
PD-1	PE	BD
IFN-γ	PerCP Cy5.5	BD

The intracellular detection of FoxP3 was performed using a FoxP3 staining kit (eBioscience, San Diego, CA) according to the manufacturer’s protocols. For intracellular IFN-γ staining, cells were stimulated for 4 h with 100 ng/ml phorbol myristate acetate, 1 µg/ml ionomycin and 0.7 µg/ml monensin. Cells were fixed in 1% PFA for 15 min. and resuspend in 0.5% PBS/BSA until acquiring by cytometer (LSRFortessa, BD). Cell population analysis was performed with FlowJo.

### Immunofluorescence staining

Immunofluorescence staining was performed on mouse brain vibratome sections as described earlier^26^. Following primary and secondary antibodies are summarized in Table 2. White bars represent 50 µm.

**Table 2:** Immunofluorescence staining.

Antigen/Primary Antibody	Origin	Secondary Antibody	Origin
GLUT-1	Millipore	Donkey Anti-rabbit Alexa 555	Life Technologies
GLUT-1	Abcam	Donkey Anti-mouse Alexa 555	Life Technologies
GLUT-1	Santa Cruz	Donkey Anti-goat Alexa 555	Life Technologies
Gal-1	Peprotech	Donkey Anti-rabbit Alexa 647	Life Technologies
F4/80	Life Technologies	Donkey Anti-rat Alexa 488	Life Technologies
MHCII	Thermo Scientific	Donkey Anti-rat Alexa 488	Life Technologies
Ki67	Abcam	Donkey Anti-rabbit Alexa 647	Life Technologies
MRC-1	R&D Systems	Donkey Anti-goat Alexa 488	Life Technologies
γ-H2AX	Cell Signaling Technology	Donkey Anti-rabbit Alexa 555	Life Technologies
Caspase-3	Abcam	Donkey Anti-rabbit Alexa 555	Life Technologies

Aqcuisition was performed on Leica SP8 confocal microscope and analyzed via Adobe Photoshop and ImageJ Using a 25x water immersion objective.

Quantitative assessment of vessel diameter was performed by measuring at least 12 vessel diameters in 3 independent pictures per mouse at day 20 post tumor inoculation.

### RT-qPCR

mRNA analysis on siRNA treated tumor biopsies were processed as described earlier^26^.Total RNA was isolated, and PCR reaction was prepared for GAPDH as housekeeping gene, and *foxP3* as gene of interest (Forward: ccc agg aaa gac agc aac ctt, Reverse: ttc tca caa cca ggc cac ttg, Taqman Probe: atc cta ccc act gct ggc aaa tgg agt c).

### Correlation between T cell-associated genetic signature and LGALS1

The metagene associated with Th1, Treg or CTLs^31, 33^ were derived from our previously published analysis where GBM-tailored T cell-metagenes were established^12^. Th1/Treg and CTL/Treg ratios were then generated based on these datasets, using the TCGA GBM Exome sequencing data. The correlation of *LGALS1* was analyzed with respect to these Th1/Treg or CTL/Treg metagene-ratios in TCGA GBM patient datasets to generate a (Pearson’s) correlation. Data retrieved from Project Betastasis webplatform.

### Prognostic impact of T cell-associated genetic signatures and *LGALS1*

The differential (exome sequencing-derived) expression levels of Th1-associated metagene^12^, CTL-associated metagene^12^ or *LGALS1* and associated clinical survival information (overall survival or OS) was retrieved and analyzed for the TCGA GBM patient data-set (n = 349)^49, 50^ using the Project Betastasis web-platform. These platforms stratified the respective patients on the basis of the median gene expression profile into two risk-groups i.e. high risk or low risk^33^. The respective patient risk groups were plotted with respect to OS to generate Kaplan-Meier curves using the Graphpad Prism software^51^. Hazard ratio (and its 95% confidence interval) and log-rank (Mantel-Cox) P values were calculated (statistical significance set at p < 0.05)^33^. Patients surviving beyond the follow-up thresholds were censored.

## Electronic supplementary material

Supplementary information
