# Supplementary material for: Sensitization of glioblastoma tumor micro-environment to chemo- and immunotherapy by Galectin-1 intranasal knock-down strategy
Source: Sci Rep. 2017 Apr 27;7:1217. doi: 10.1038/s41598-017-01279-1 (PMC5430862; doi:10.1038/s41598-017-01279-1)
Supplement: Supplementary file 1 — Supplementary information [file 41598_2017_1279_MOESM1_ESM.pdf]

Supplementary Data

TITLE

Sensitization of glioblastoma tumor micro-environment to chemo- and immunotherapy by Galectin-1 intranasal knock-down strategy

AUTHORS

Matthias Van Woensel (1,2,*) & Thomas Mathivet (3,4,*), Nathalie Wauthoz (2), Rémi Rosière (2), Abhishek D. Garg (5), Patrizia Agostinis (5), Véronique Mathieu (6), Robert Kiss (6), Florence Lefranc (7), Louis Boon (8), Jochen Belmans (9), Stefaan W. Van Gool (10), Holger Gerhardt (3,4), Karim Amighi (2), Steven De Vleeschouwer (1,11)

Affiliations

(1) Research Group Experimental Neurosurgery and Neuroanatomy, KU Leuven, Herestraat 49, Leuven 3000, Belgium;

(2) Laboratoire de Pharmacie Galénique et de Biopharmacie, Université libre de Bruxelles (ULB), Boulevard du triomphe CP207, Brussels 1050, Belgium;

(3) Vascular Patterning Unit, VRC, VIB, Herestraat 49 box 912, Leuven 3000, Belgium

(4) Vascular Patterning Laboratory (Vesalius Research Center), Department of Oncology, KU Leuven, Herestraat 49 box 912, Leuven 3000, Belgium

(5) Cell Death Research & Therapy (CDRT) Lab, Department of Cellular and Molecular Medicine, KU Leuven, Herestraat 49, Box 802, Leuven, 3000 Belgium;

(6) Laboratoire de Cancérologie et de Toxicologie Expérimentale, Université libre de Bruxelles (ULB), Boulevard du triomphe CP205/01, Brussels 1050, Belgium;

(7) Department of Neurosurgery, Erasmus University Hospitals, Route de Lennik 808-1070, Brussels 1050, Belgium;

(8) EPIRUS Biopharmaceuticals, BV, Yalelaan 46, Utrecht 3584, The Netherlands;

(9) Laboratory of Pediatric Immunology, KU Leuven, Herestraat 49 box 811, Leuven 3000, Belgium;

(10) Medizinische Leitung der Translationalen Onkologie, Immunologisches Onkologisches Zentrum, Köln , Germany;

(11) Department of Neurosurgery, University Hospitals Leuven, Herestraat 49 box 7003, Leuven 3000, Belgium.

* Authors share first authorship; equal contribution

Supplementary Figure 1

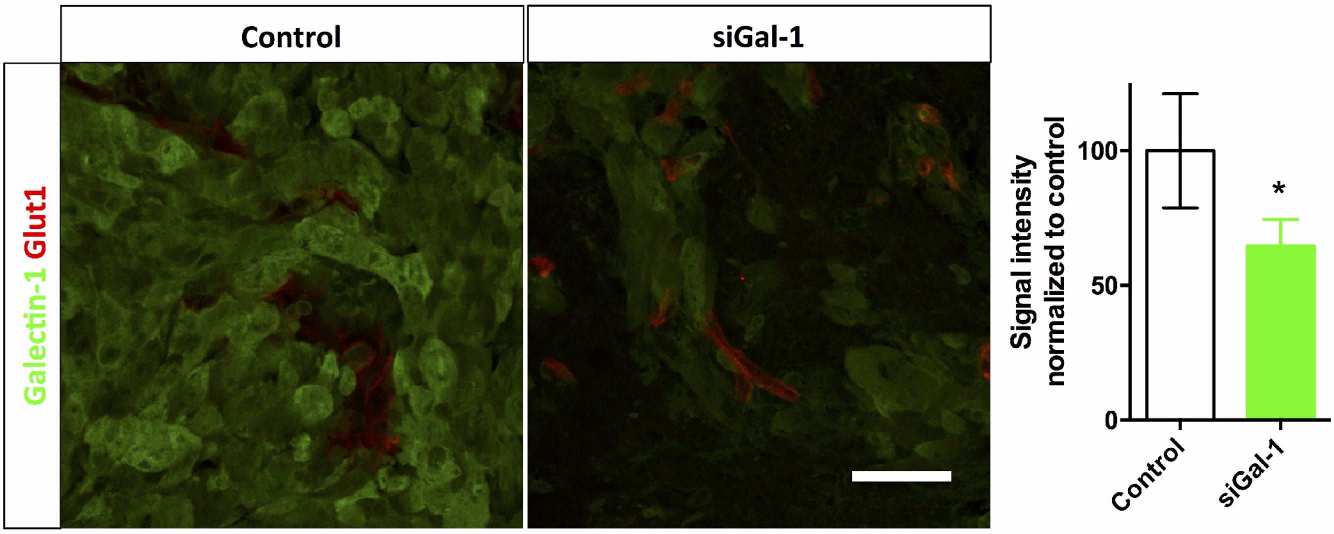

Supplementary Figure 1: Intratumoral Gal-1 reduction. Mice were inoculated with 0.5 x 10^6 GL261 cells which induces a lethal GBM after 15-20 days. Representative pictures of late stage tumors are shown for control mice or mice treated with siGal-1 as described in Figure 1A. Green: Gal-1 and Red: Glut-1. On the right side, the quantification of Gal-1 intensity in at least 3 slices/mouse, demonstrates a significant reduction of Gal-1 in the tumor. (n=5/group, * = $p<0.05$, unpaired t – test). Scale bar: 100µm.

Supplementary Figure 2

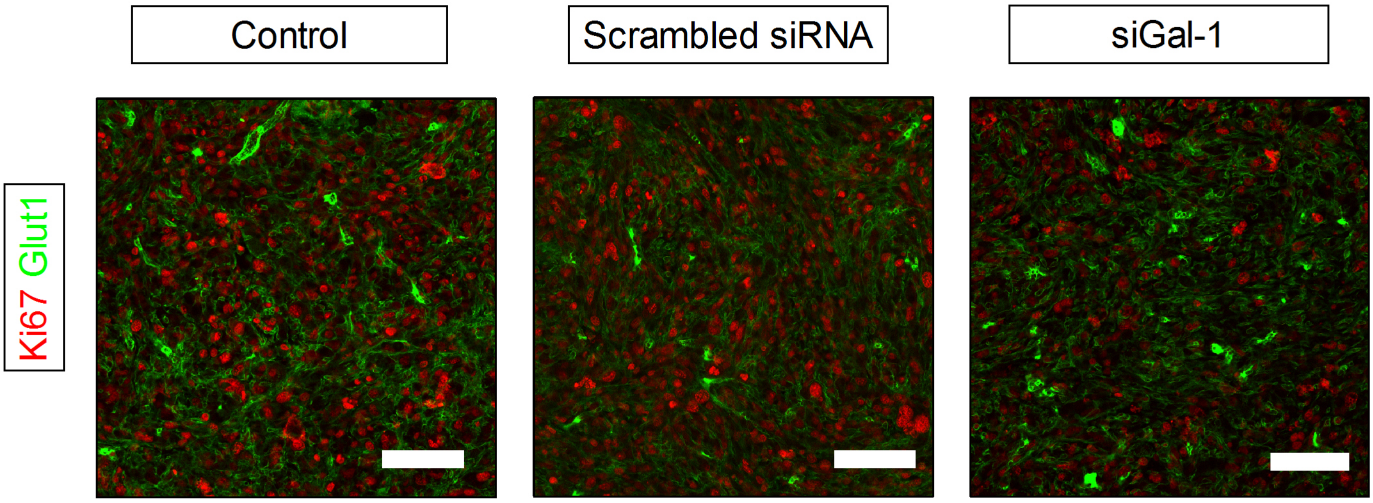

Supplementary Figure 2: Ki67 staining pictures. Mice were inoculated with 0.5 x 10^6 GL261 cells which induces a lethal GBM after 15-20 days. Representative pictures of late stage tumors are shown for control, untreated mice; mice treated with scambled siRNA loaded chitosan nanoparticles, or mice treated with siGal-1. Scale bar: 250µm.

Supplementary Figure 3

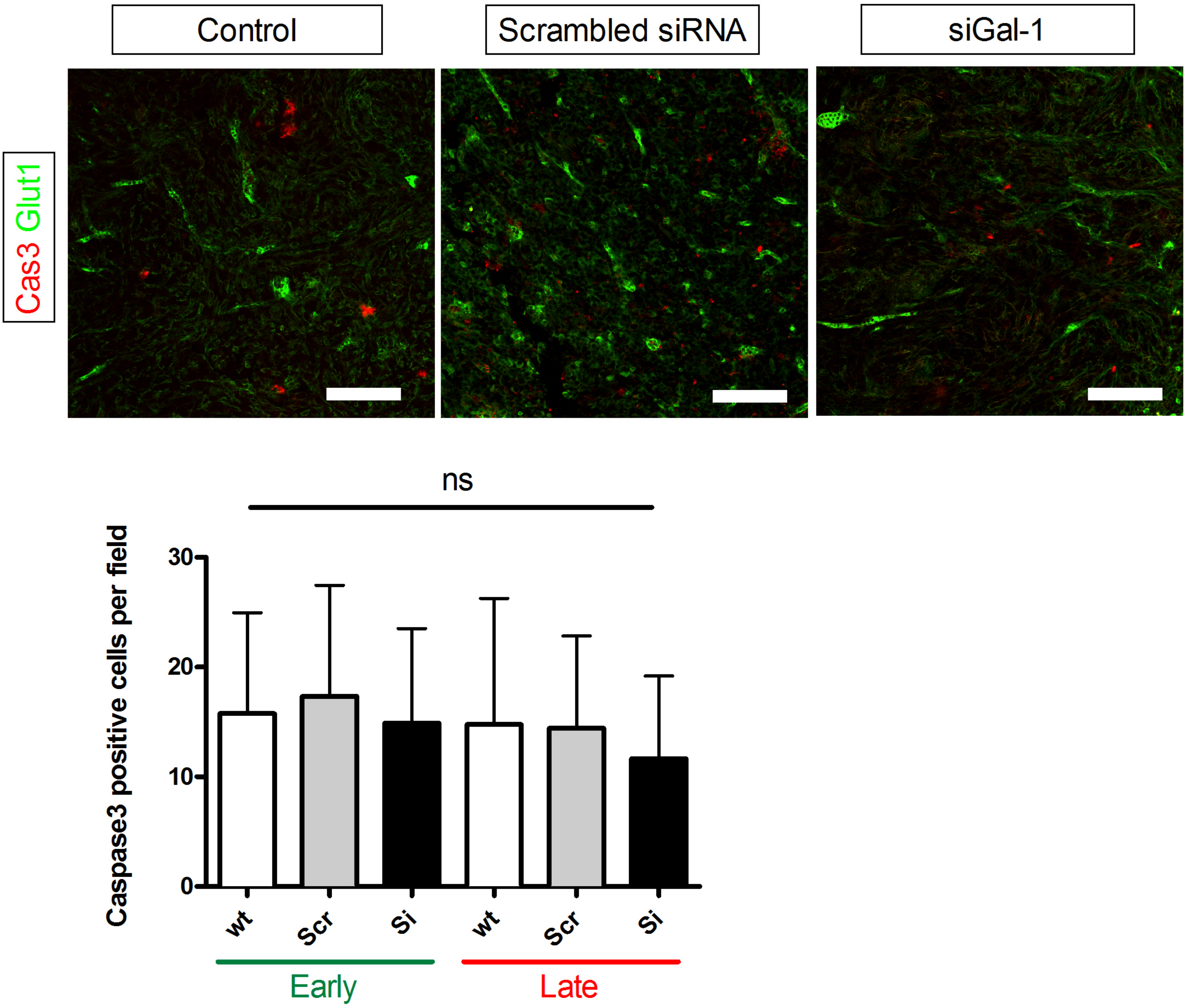

Supplementary Figure 3: Cas3 staining pictures. Mice were inoculated with 0.5 x 10^6 GL261 cells which induces a lethal GBM after 15-20 days. Representative pictures of late stage tumors are shown for control, untreated mice; mice treated with scambled siRNA loaded chitosan nanoparticles, or mice treated with siGal-1. Moreover, quantification is provided of multiple sections/mice (n=4/group, at least 3 independent fields/mouse). Scale bar 200µm.

Supplementary Figure 4

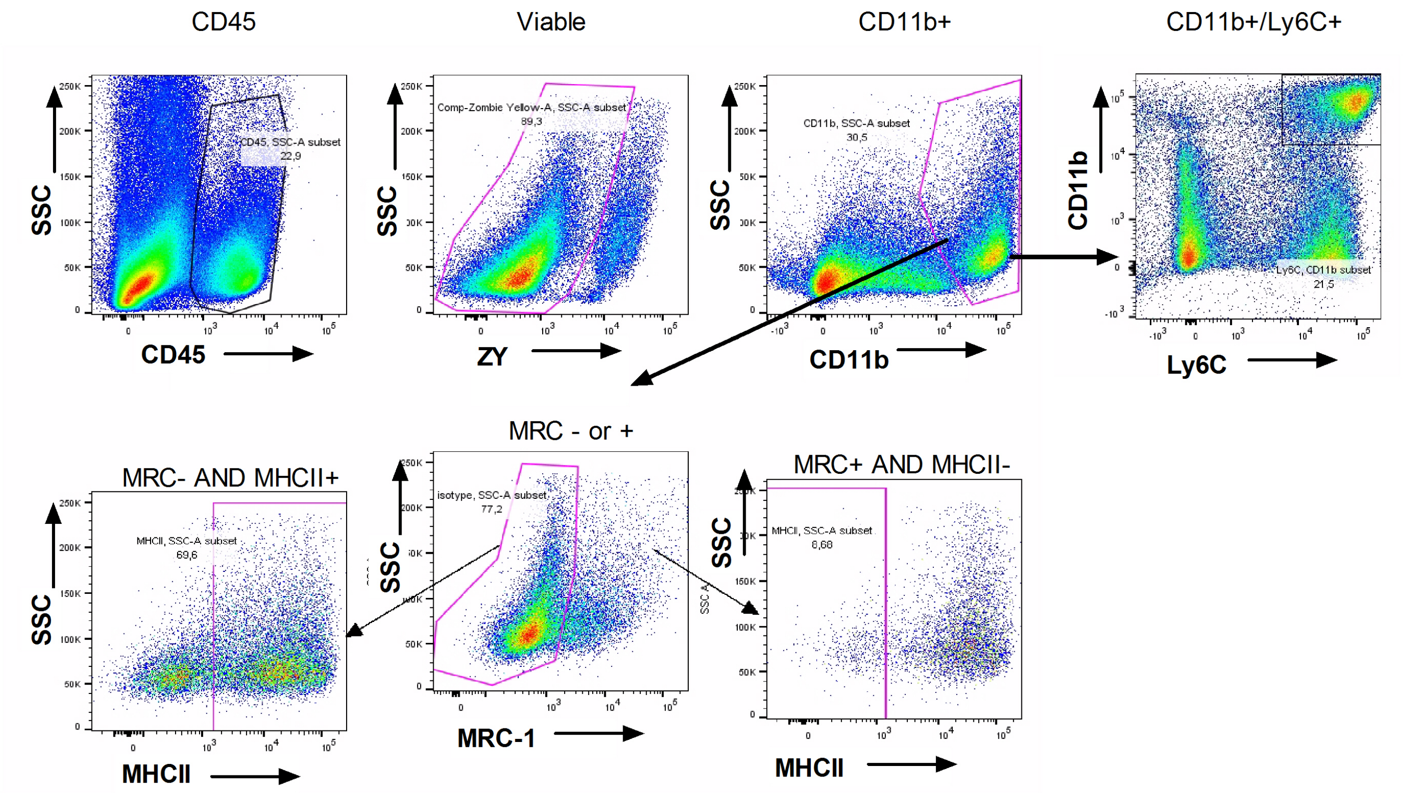

Supplementary Figure 4: Gating strategy for myeloid cell population. Flow cytometry was performed on isolated mononuclear brain infiltrating cells of mice and gated for CD45 positive (leukocytes), viable cells (ZY negative), and CD11b positive (myeloid). Subsequently, we looked either into Ly6C for monocytic MDSCs and Ly6G for granulocytic MDSCs. Futhermore, we also analysed the CD11b positive cells for MRC1 expression (middle, down row), and we monitored for MRC1 negative cells for MHCII positive (M1 macrophage phenotype), on the other side, we monitored the MRC1 positive cells for MHCII negative (M2 macrophage phenotype).

Supplementary Figure 5

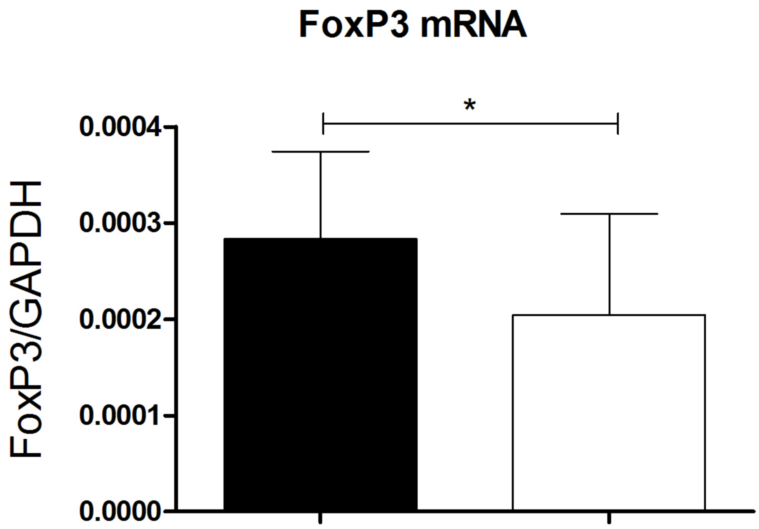

Supplementary Figure 5: mRNA analysis for FoxP3. Mice were inoculated with 0.5 x 10^6 GL261 cells which induces a lethal GBM after 15-20 days. RT-qPCR was performed on isolated GBM specimen of mice that were left untreated, or treated with siGal-1 on day 4, 8, 12 and 15 after tumor inoculation, and brains were isolated at day 20, which revealed a significant reduction for *foxP3* transcription factor expression (n = 10/group, * = $p < 0.05$).

Supplementary Figure 6

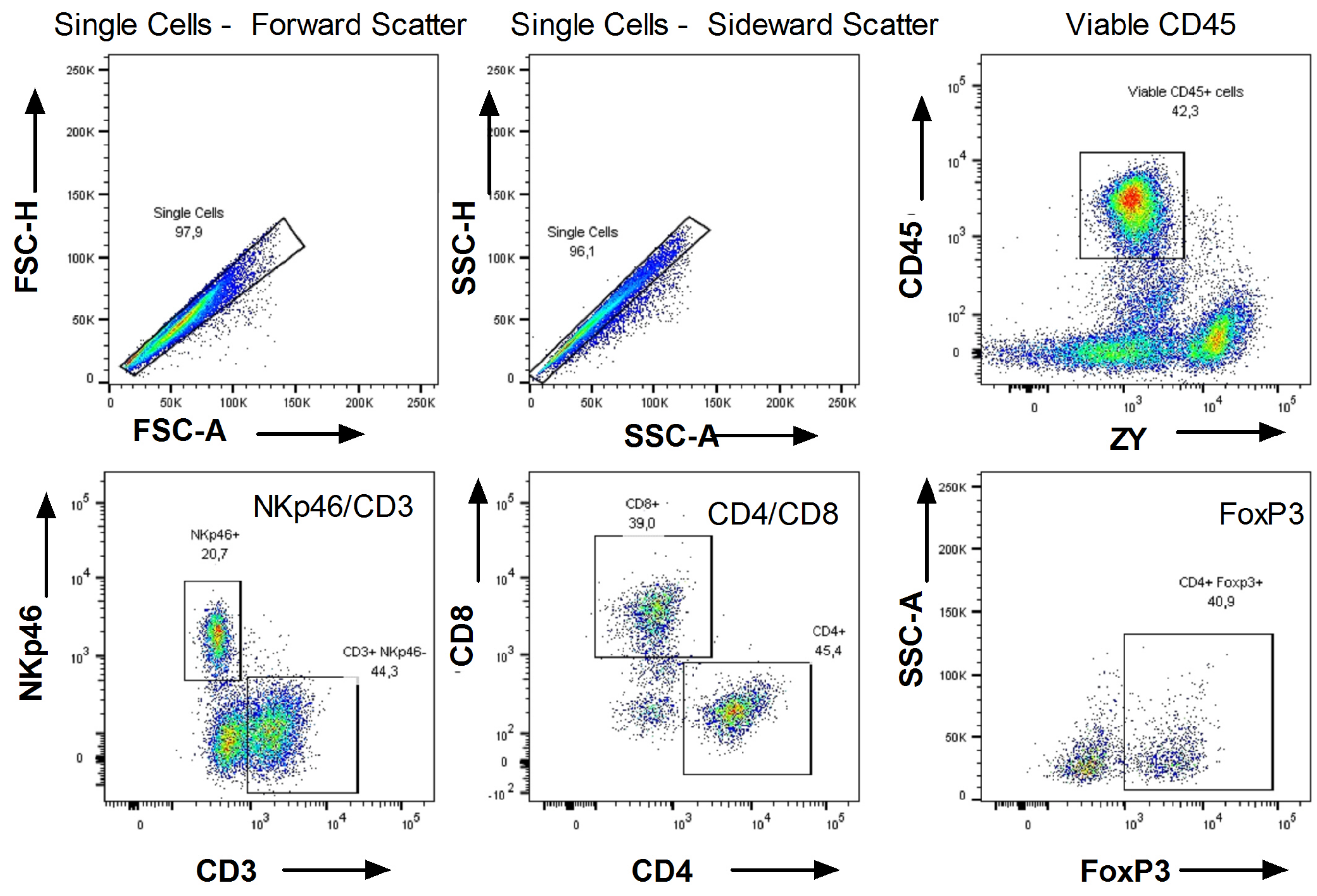

Supplementary Figure 6: Gating strategy for Treg. Flow cytometry was performed on isolated mononuclear brain infiltrating cells of mice and gated for single cells (via SSC and FSC), CD45 positive (leukocytes), viable cells (ZY negative), and CD3 positive (lymphoid). Subsequently, we looked into CD4 and CD8 for T cells, and within the CD4 gate, we monitored the expression of FoxP3 expression.

Supplementary Figure 7

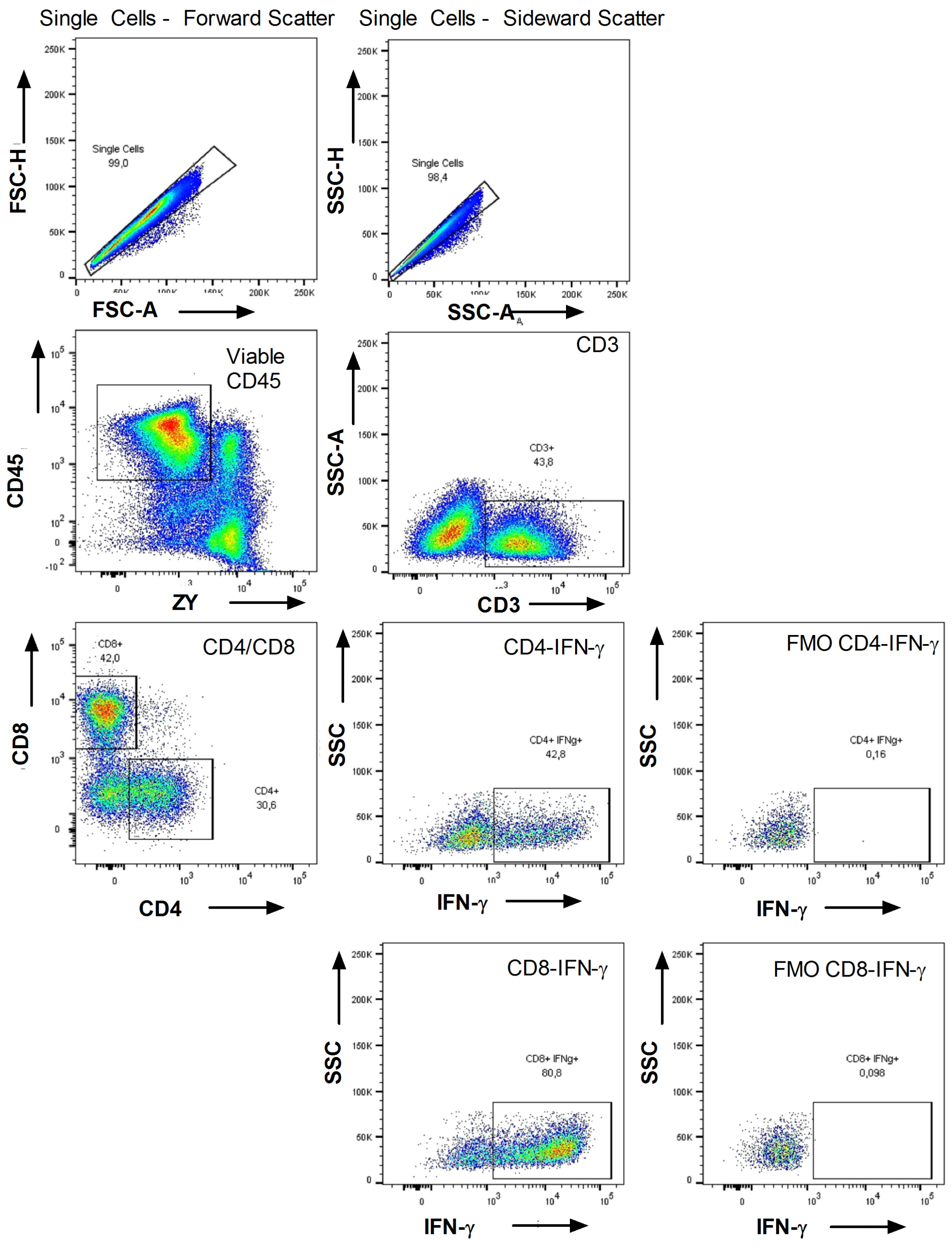

Supplementary Figure 7: Gating strategy for Lymphoid cell populations. Flow cytometry was performed on isolated mononuclear brain infiltrating cells of mice and gated for single cells (via SSC and FSC), CD45 positive (leukocytes), viable cells (ZY negative), and CD3 positive (lymphoid). Subsequently, we looked into CD4 and CD8 for T cells, and for both cell populations, we monitored the expression of IFN-γ expression, as guided by Fluorescence Minus One, to determine the proper gating strategy.
